# Supplementary material for: Olfactory modulation of barrel cortex activity during active whisking and passive whisker stimulation
Source: Nat Commun. 2022 Jul 2;13:3830. doi: 10.1038/s41467-022-31565-0 (PMC9250522; doi:10.1038/s41467-022-31565-0)
Supplement: Supplementary file 1 — Supplementary Information [file 41467_2022_31565_MOESM1_ESM.docx]

**Supplementary Materials**

*Renard et al*., Olfactory modulation of barrel cortex activity during active whisking and passive whisker stimulation


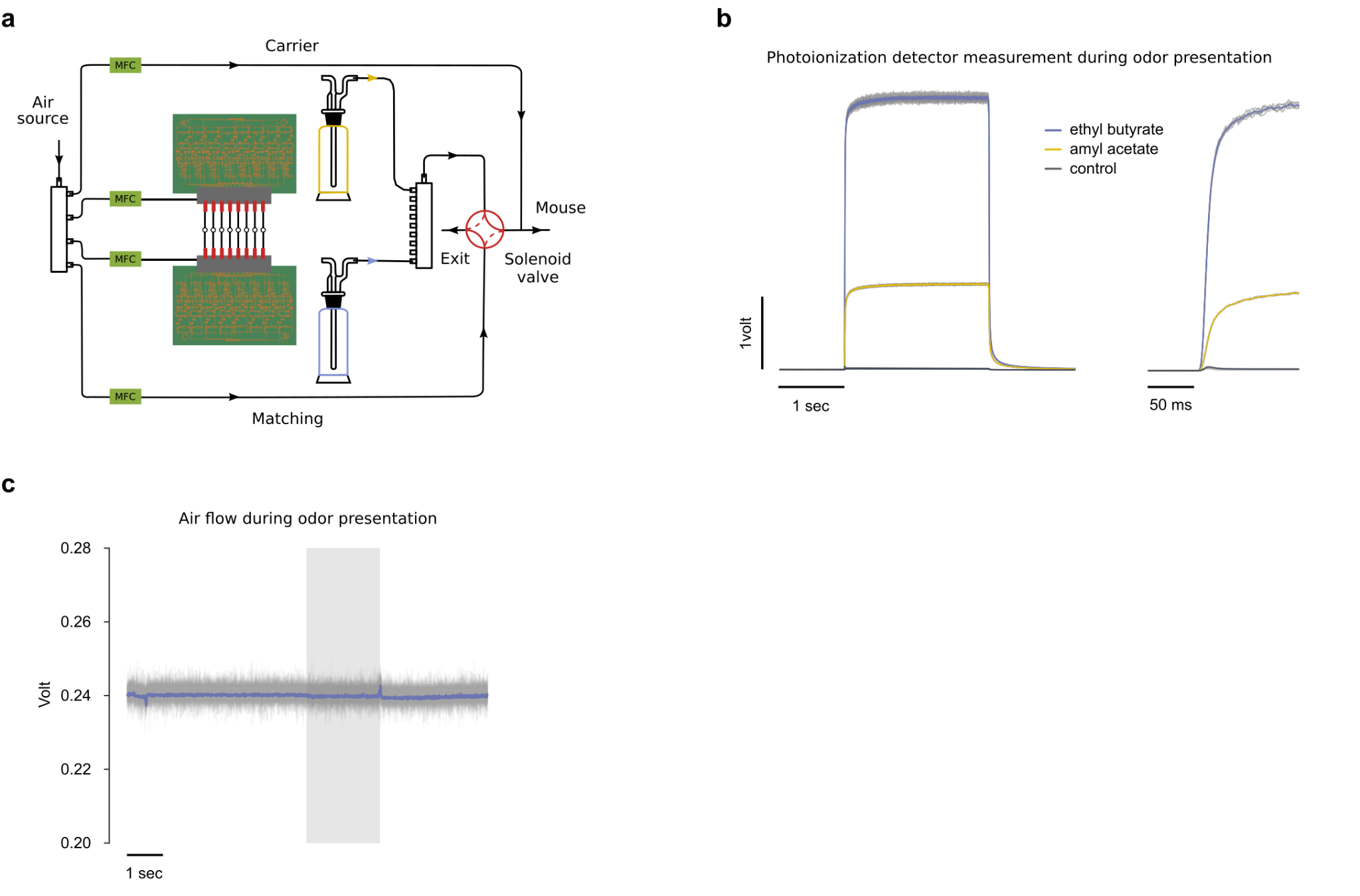


**Supplementary figure 1.** Design and calibration of the custom-built olfactometer. **a** The olfactometer was constructed with chemically inert 1/16” and 1/32” inner diameter polytetrafluoroethylene tubing. The flow rates of four independent streams were regulated by mass flow controllers (MFC; SFC5400, Sensirion). Two MFC’s (250 ml/min each) controlled the flow producing odorized air by passing through 20 ml of odor solutions prediluted at 0.1% in mineral oil and contained in small bottles (Wilmad ML-1490-702, SP Scienceware). Two MFC’s controlled the carrier and matching streams (500 ml/min each). The carrier stream was constantly delivering 500 ml/min to the mouse. A shuttle valve was used to switch between matching and odorized streams with precise timing. The output flow to the animal was constant at 1 L/min. Valves to an empty bottle were actuated during trials without odors. **b** Photoionization detector (PID) measurements showing temporal precision of odor presentation. Full trace (left) and magnification of the onset phase (right). The PID was placed at the location of the mouse’s snout. Gray traces correspond to single traces with n=10 for each stimulus. **c** Air flow measurement at olfactometer output during odor delivery. Blue trace represents mean over odor presentations for all pure odors and mixtures used, including the control. Gray traces correspond to single traces with n=10 for each stimulus. Shading indicates odor presentation. Flow change at 0.5 and 7 sec correspond to flow adjustment at opening and closing of the two odor delivery MFC’s, nonspecific to odor presence and identity. Note that this change in air flow is three orders of magnitude smaller than the change elicited by a sniff (e.g. see breathing trace in Fig. 7 d for comparison). Note also that the solenoid valve switch at stimulus onset does not disrupt the air flow. These measurements were performed with the microbridge mass air flow sensor (Honeywell AWM3300V, Morris Plains, NJ) used for breathing monitoring.


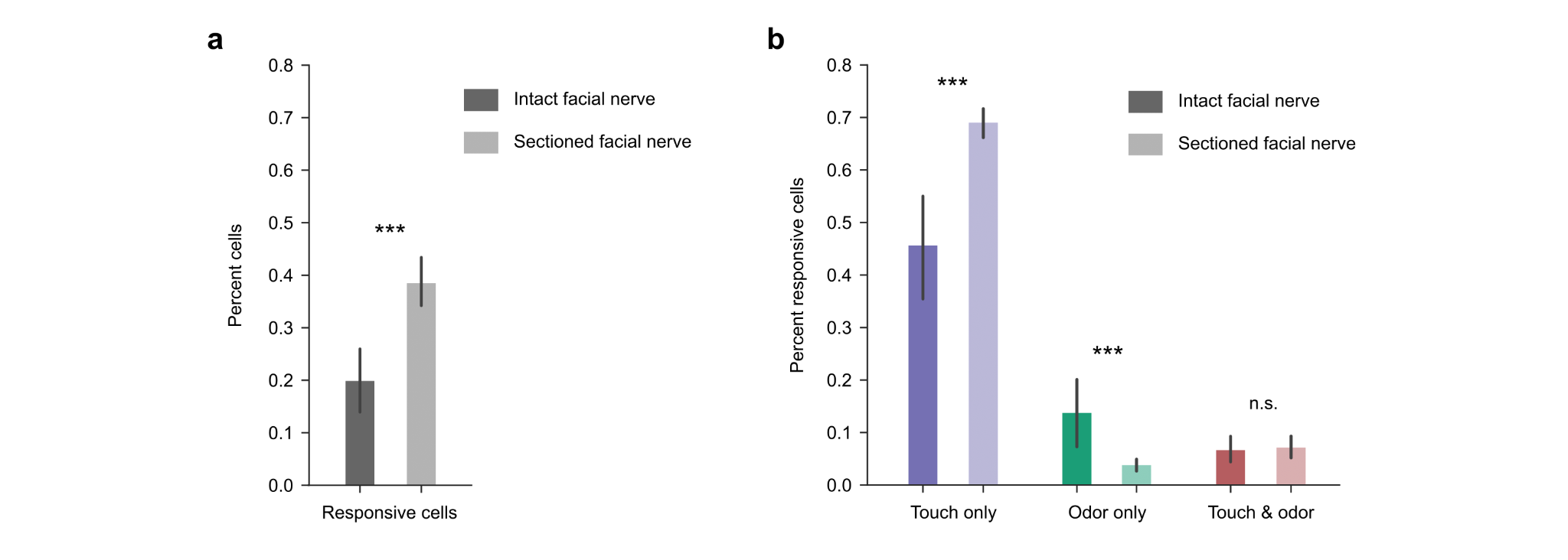


**Supplementary figure 2. Proportion of responsive cells and distribution of response categories in the active and passive contexts.** **a.** Proportion of neurons responding to at least one of the nine stimulation conditions across sessions for the active context (dark) and the passive context (light). Significance threshold = 5%; Kruskal-Wallis test. Responsiveness was significantly higher in the passive context due to the sweep of tactile stimulation applied to the whiskers (p = 0.005; Mann-Whitney U test, n=19 in passive and 20 in the active context). **b.** Distribution of responsive cells responding only to tactile gratings (blue), only to odors (green) and to both tactile gratings and odors (red). A cell was classified in the touch only category if responding significantly to tactile gratings compared to blank stimuli but not responding significantly to odors (significance threshold = 5%; Mann-Whitney U test, n=19 in passive and 20 in the active context). Similarly, a cell was classified in the odor only category if it responded significantly to odors but not to tactile gratings. The touch and odor category was defined as the intersection of the cells responding significantly to tactile gratings and to odors. The proportion of touch only cells was significantly higher in the passive context, while the proportion of odor only cells was lower and the proportion of cells responding to both was not significantly different (p = 0.002, p = 0.002, p = 0.77, respectively; Mann-Whitney U test, n=19 in passive and 20 in the active context). Error bars indicate 95% confidence intervals for the mean throughout the figure. All tests are two-sided.


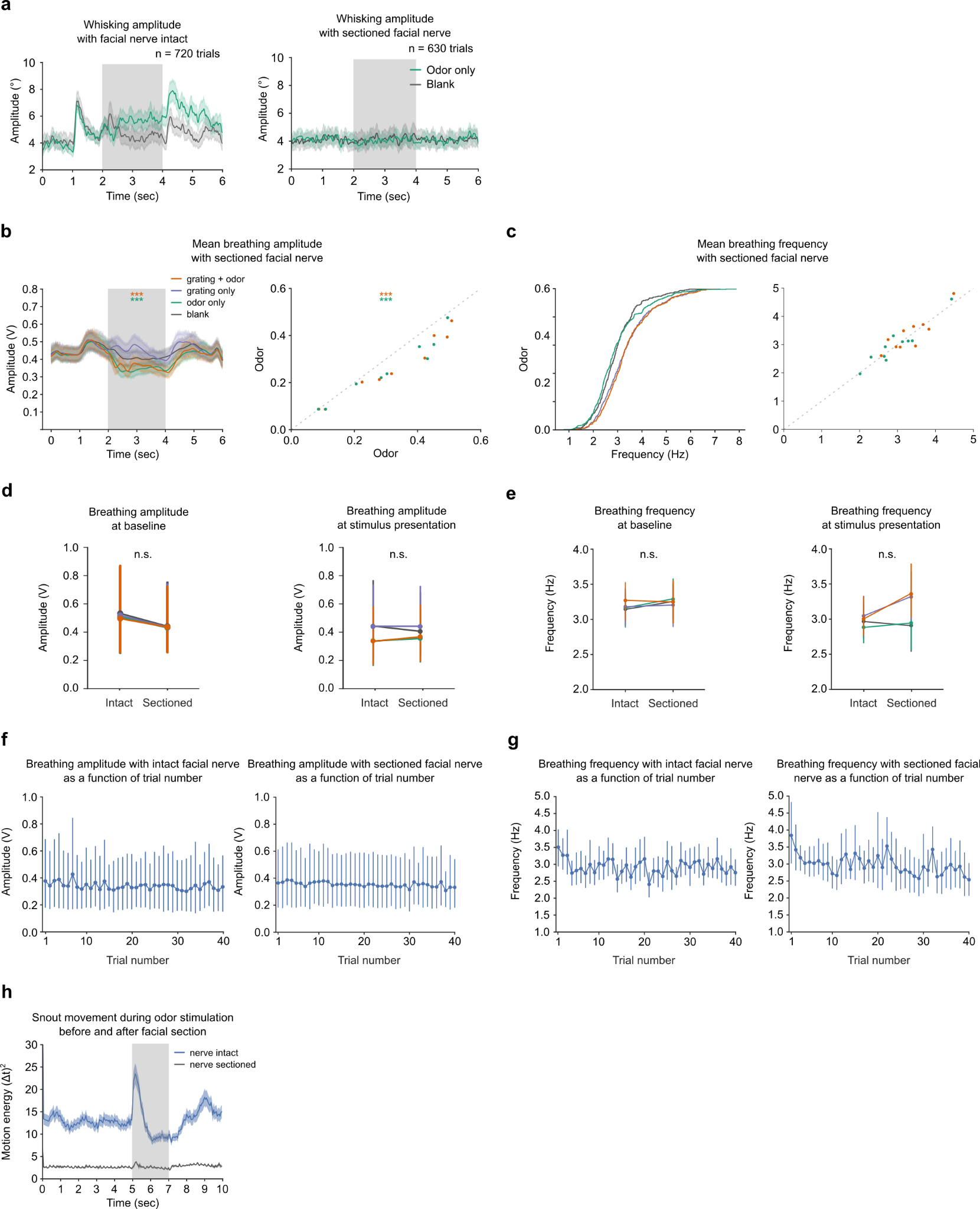


**Supplementary figure 3. Sectioning the buccal and marginal mandibular branches of the facial nerve abolishes whisking and does not impact breathing. a** Left: trial-averaged whisking amplitude within sessions for trials without tactile gratings for mice with intact facial nerves (active context). Shading indicates 95% CI; n = 720 trials from 18 sessions and 10 mice. Right: analogous to *a* for mice with sectioned facial nerves (passive context); n = 630 trials from 9 sessions and 5 mice (some sessions from the passive context were not included as videos of the whiskers were not recorded for all sessions in this context). The baseline of 4° in amplitude is the result of an imprecision in whisker detection from frame to frame and from residual motion of the whiskers due to facial motion that is preserved after facial nerve resection. **b** Left: mean breathing amplitude within sessions across time for the passive context. Right: scatter plot of mean breathing amplitude between sessions at stimulus presentation. **c** Right: cumulative distribution of breathing frequency within sessions for the passive context. Left: scatter plot of mean breathing frequency between sessions at stimulus presentation. In b and c, n = 19 sessions from 6 mice; *p<0.05, **p<0.01, ***p<0.001; no star p>0.05, Wilcoxon rank-sum test. Wilcoxon signed-rank test; see **Supplementary Table 3** for detailed statistics. **d** Comparison of breathing amplitude at baseline (right) and stimulus presentation (left) showing no significant difference between the active and passive contexts. **e** Same as *d* for breathing frequency; Wilcoxon signed-rank test (in d-e, n=19 in the passive and 20 in the active context). **f** Trial-averaged breathing amplitude as a function of trial number between the active (left) and passive contexts (left) during odor presentations. Error bars indicate 95% CI over sessions. **g** Same as *f* for breathing frequency. Note a ~1 Hz increase in breathing frequency at the first odorant presentation. In f-g, n=19 in the passive and 20 in the active context. **h** Motion energy of the snout showing that facial nerve resection strongly abolishes snout movement. Motion energy was quantified as the square of the pixel difference between consecutive frames for a region of interest defined on the snout; n = 660 trials from 2 sessions in 2 mice. Error bars and bands indicate 95% confidence intervals for the mean throughout the figure. All tests are two-sided.

**
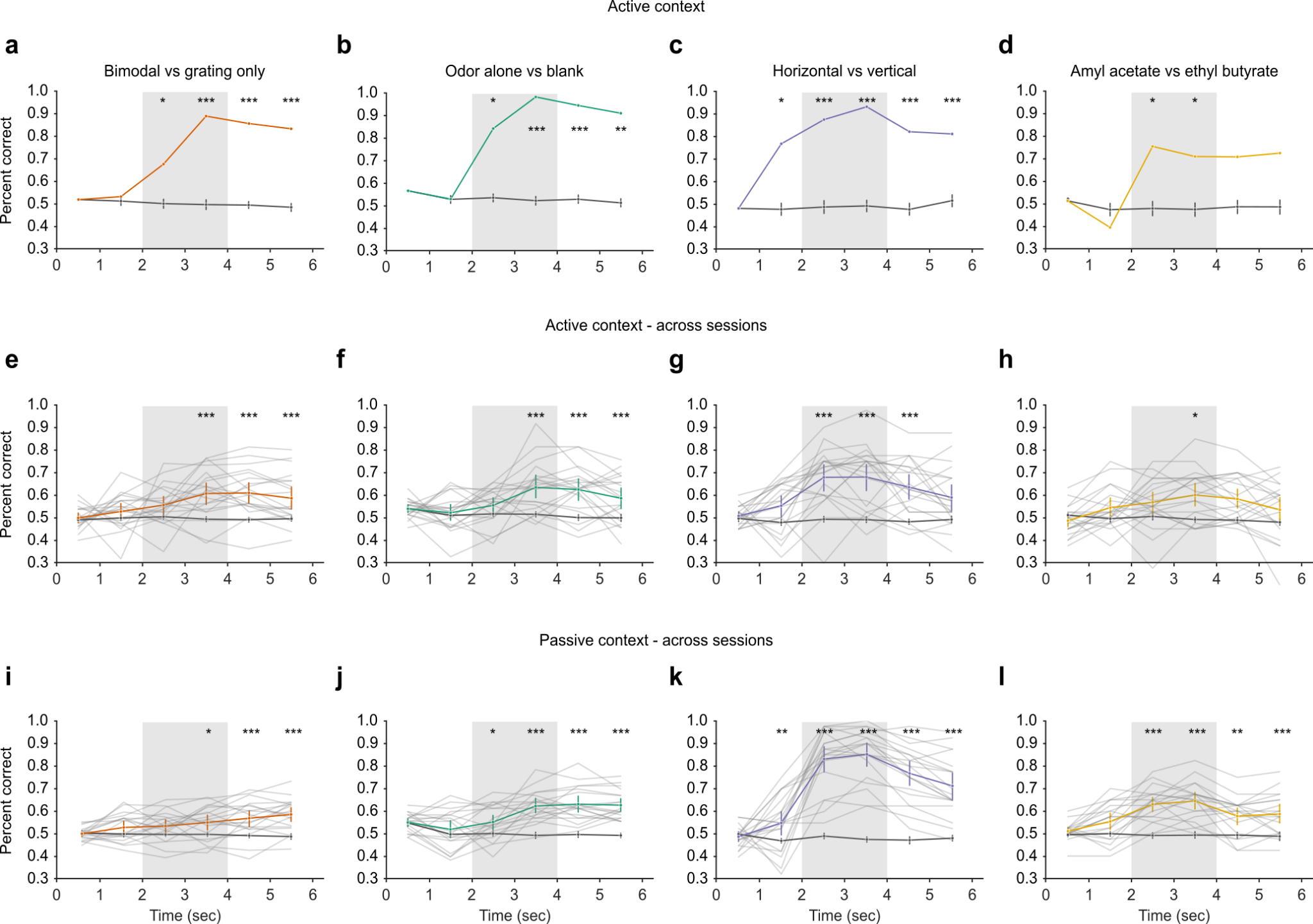
**

**Supplementary figure 4.** **Olfactory information is also present in the active context in S1 and across sessions in both contexts.** **a-d** Accuracy of centroid classifier decoding of stimulus from S1 activity in the active context averaged over 1 sec time bins; analogous to **Fig. 6 a-d**. Performance for shuffled labels is shown in gray. P-values were obtained as the location of the mean accuracy in a distribution of 1000 shuffles; *p<0.05, **p<0.01, ***p<0.001 (two-sided, no correction for multiple testing). Shading indicates stimulus presentation. **e-h** Analogous to **a-d** with decoding performance across sessions in the active context. Each data point is the average of a 20-fold stratified cross-validation with data from single sessions. Significance was assessed by comparing the mean accuracy of real and shuffled data with n = 20 and n = 19 sessions in the active and passive contexts, respectively (two-sided Wilcoxon signed-rank test). Gray lines indicate single session performance. **i-l** Analogous to **e-h** in the passive context. Note that the data was z-scored to achieve significant performance in the results presented in this figure, which was not the case in **Fig. 6**. Error bars indicate 95% confidence intervals for the mean throughout the figure.


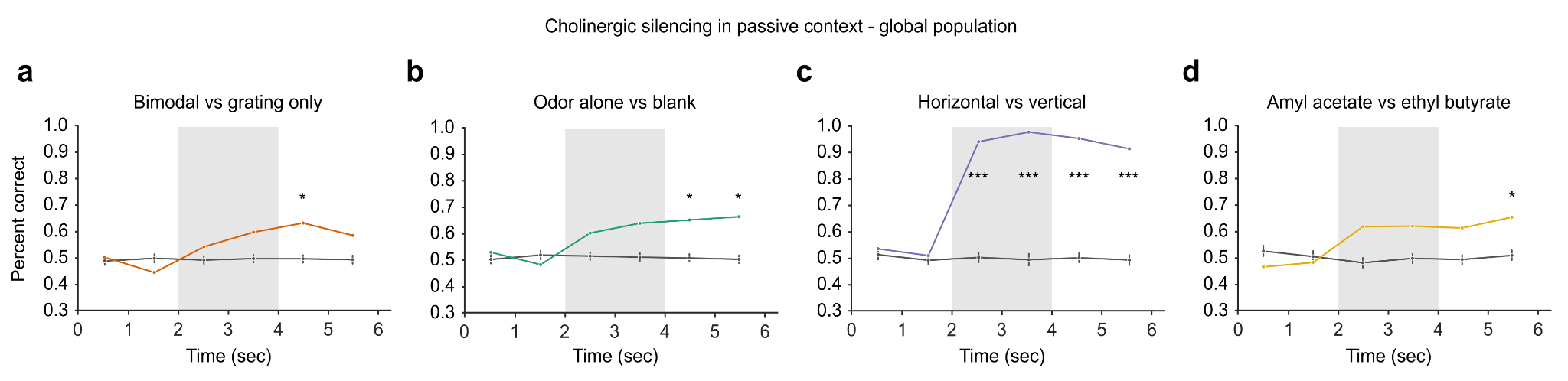


**Supplementary figure 5. Olfactory information is present during silencing of cholinergic input to S1. a-d** Accuracy of centroid classifier decoding of stimulus during silencing of cholinergic inputs from S1 activity in the passive context averaged over 1 sec time bins; analogous to **Fig. 6 a-d**. Performance for shuffled labels is shown in gray (Error bars indicate 95% confidence intervals for the mean). P-values were obtained as the location of the mean accuracy in a distribution of 1000 shuffles; *p<0.05, **p<0.01, ***p<0.001 (two-sided, no correction for multiple testing). Shading indicates stimulus presentation. The data was z-scored similarly to **Supplementary Fig. 4**.


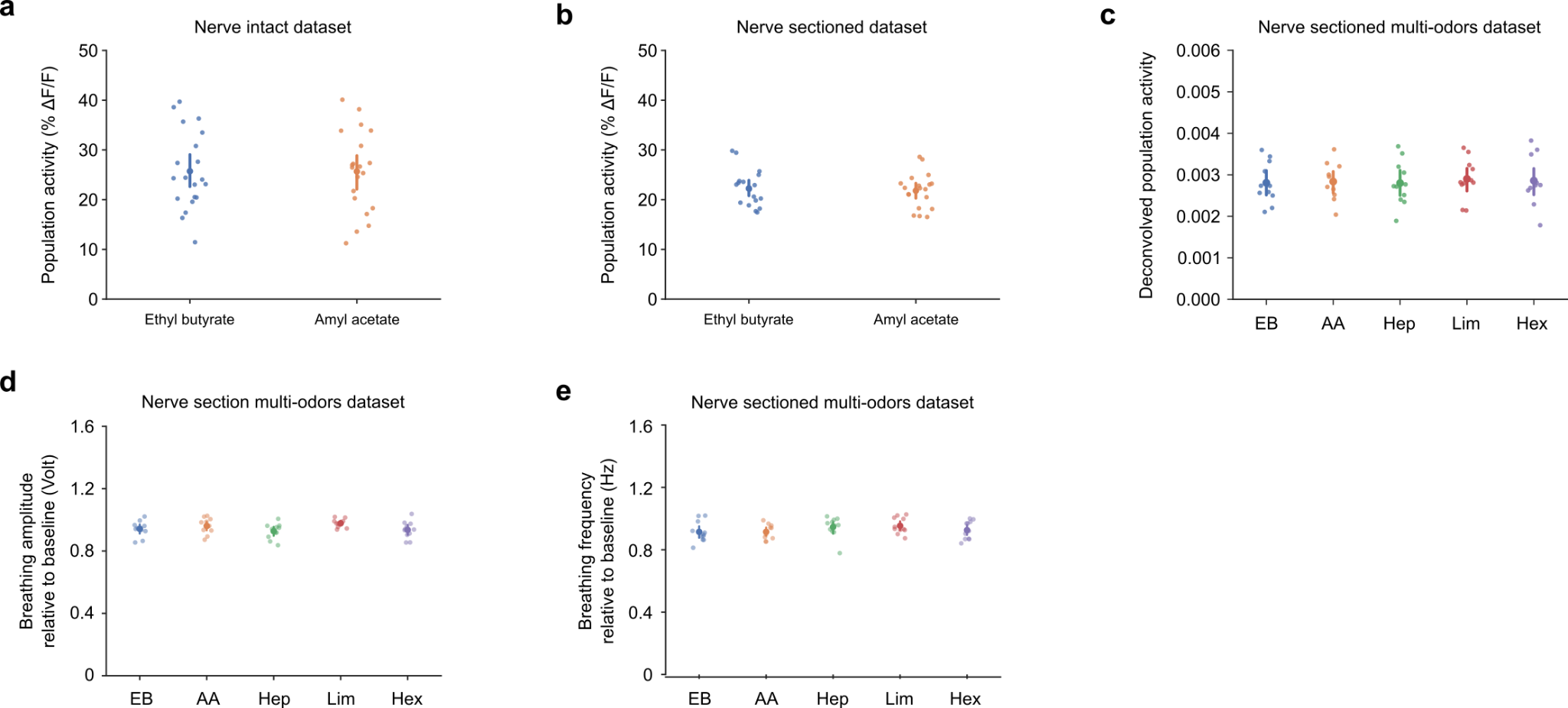


**Supplementary figure 6. Mean population firing rate, breathing and whisking do not differ across odors. a.** Mean population response for the two presented odors averaged across all active neurons during the active context (facial nerve intact). **b.** Same as *a* but for the passive context (facial nerve resected). **c.** Mean population response for the five pure odorants presented expressed as the deconvolved fluorescence signal averaged across all odor responsive neurons during the olfactory stimulation protocols in the multi-odor passive context (**Fig. 7**). **d** Average breathing amplitude during olfactory stimulation normalized to baseline for the five pure odorants presented. **e** Same as *d* for breathing frequency.


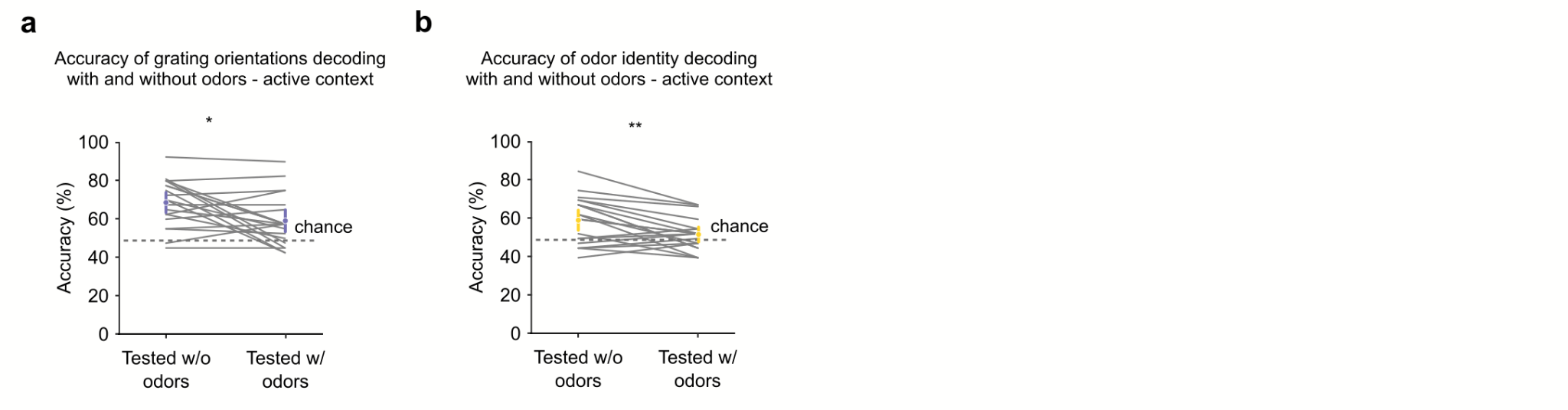


**Supplementary figure 7. Interaction between olfactory and tactile representation in the active context. a.** Accuracy of tactile grating orientation decoding (0 vs 90°) for a classifier trained on tactile responses in the absence of odors and tested in absence or presence of odor. Contrary to what we observed in the passive context (**Fig. 6**), odor presentation impacts tactile representations in the active context, putatively due to changes in whisking behavior (p=0.07, two-sided Wilcoxon signed-rank test, * indicates p<0.05,). **b.** Accuracy of odor identity decoding (Amyl Acetate vs Ethyl Butyrate) for a classifier trained on odor responses in the absence of tactile stimulus and tested in absence or presence of tactile stimulus (p=0.005, two-sided Wilcoxon signed-rank test, ** indicates p<0.01.). Tactile stimuli impact the odor representation in the active context like in the passive context (**Fig. 6**).


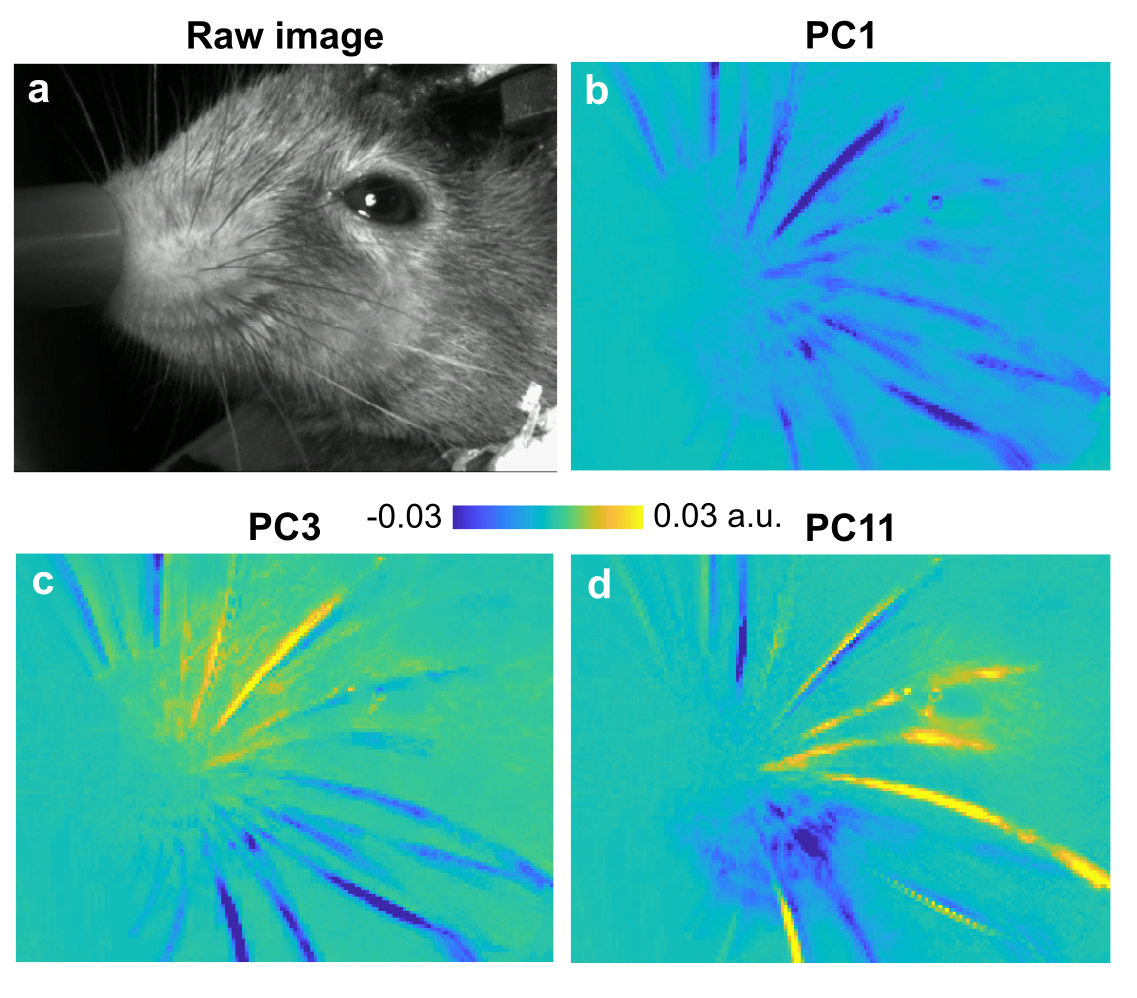


**Supplementary figure 8. Principal components of facial behavior. a.** Sample frame of a video recording of facial movements made during olfactory stimulation and two-photon calcium imaging. The tube on the mouse’s nose is the flow sensor for sniff monitoring. **b-d.** Weights of the three example principal components (PC) displayed in **Fig. 7f**. By visual inspection, PC1 captures global movements, PC3 captures a mode of whisker movements, and PC11 captures another mode of whisker movements as well as eyelid and jaw movements. The first 500 PCs are used for decoding of odor identity in **Fig. 7h**; using less PCs did not affect the result (**Supplementary Fig. 9**). Note that even though whisking is abolished by sectioning of the buccal and marginal mandibular branches of the facial nerve, whisker motion is still possible through their connection with facial tissue.

**
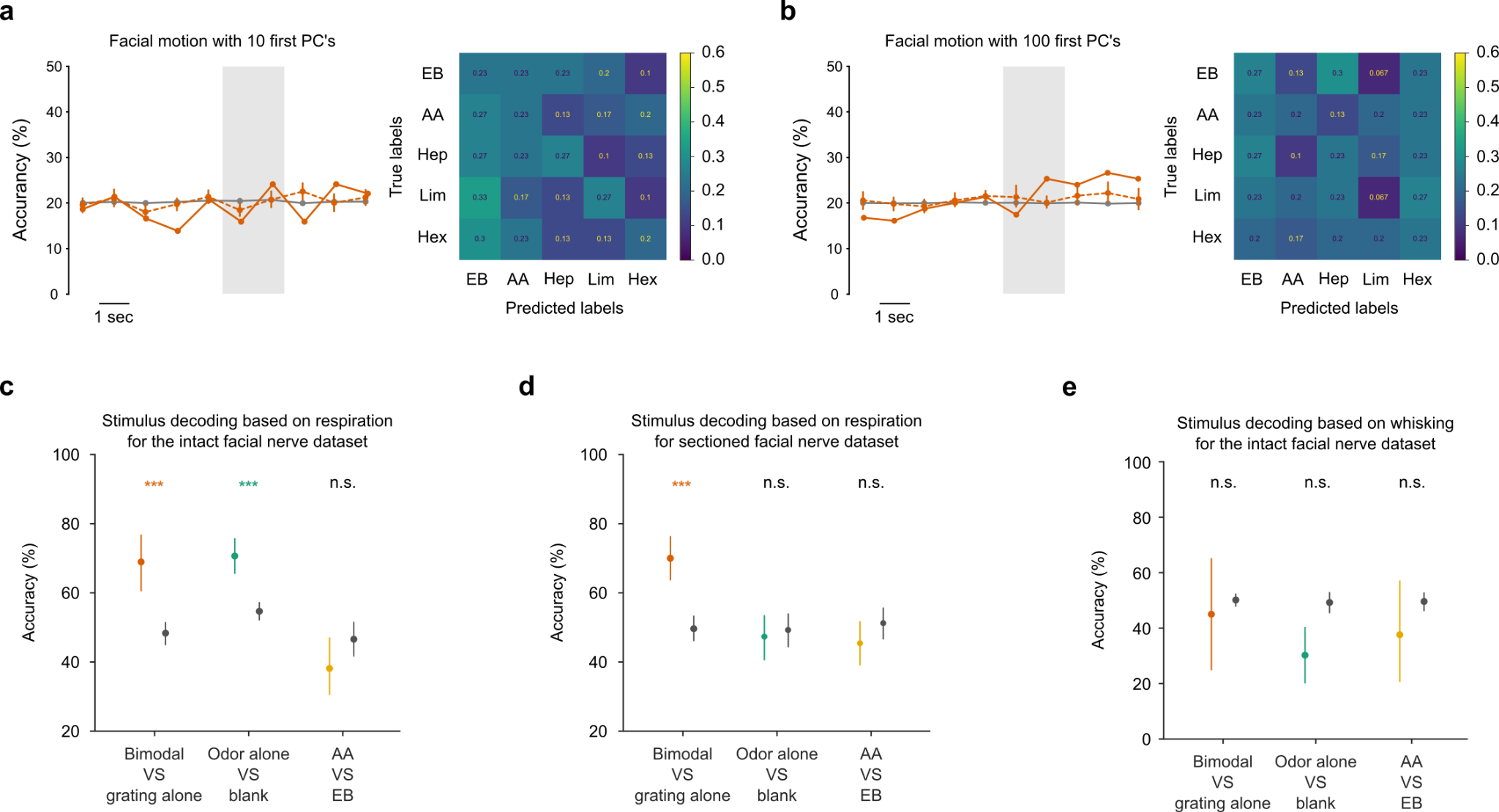
**

**Supplementary figure 9. Odor decoding based on facial movements, respiration or whisker kinematics performs poorly independent of processing parameters. a** Left: decoding accuracy of odor identity as in **Fig. 7h** but using the first 10 facial behavior PCs only. Chance level performance is obtained whether classification is done in single sessions (dashed line) or after pooling PCs of all sessions (plain line). Right: confusion matrix of the pooled-session classifier. **b** Same as *b* but with the first 100 PCs. Using less PCs did not improve decoding accuracy of odor identity based on facial behavior. **c** Accuracy of linear SVM classifiers decoding of odor presence and identity from breathing amplitude and frequency in the active context during stimulus presentation averaged over 100 ms time bins. Performance for shuffled labels is shown in gray. P-values were obtained as the location of the mean accuracy in a distribution of 1000 shuffles; *p<0.05, **p<0.01, ***p<0.001. Error bars indicate 95% CI of cross-validation iterations with n = 10 for real data and n = 100 for shuffled data. **d** Same as *f* for the passive context. **e** Same as *f* but decoding is based on whisking amplitude, setpoint and absolute curvature change in the active context.

| **Within sessions** | | | |
| --- | --- | --- | --- |
|  | | | |
|  | | | |
|  | **Condition** | **Mean (95% CI)** | **p-value** |
| **Amplitude** | Bimodal | 4.67 (4.37, 4.99) | 0.081 |
|  | Grating only | 4.31 (4.01, 4.63) |  |
|  | Odor only | 6.01 (5.60, 6.43) | 1.10^-6^ |
|  | Blank | 5.08 (4.62, 5.59) |  |
| **Setpoint** | Bimodal | 82.51 (81.81, 83.19) | 0.12 |
|  | Grating only | 81.91 (81.20, 82.62) |  |
|  | Odor only | 86.50 (85.71, 87.30) | 0.014 |
|  | Blank | 85.25 (84.34, 86.17) |  |
| **Curvature change** | Bimodal | 0.053 (0.048, 0.058) | 0.04 |
|  | Grating only | 0.048 (0.043, 0.052) |  |
|  | Odor only | 0.030 (0.028, 0.032) | 0.001 |
|  | Blank | 0.024 (0.023, 0.026) |  |
| **Between sessions** | | | |
| **Amplitude** | Bimodal | 4.65 (3.83, 5.49) | 0.042 |
|  | Grating only | 4.27 (3.62, 5.00) |  |
|  | Odor only | 6.01 (5.6, 6.43) | 1x10^-4^ |
|  | Blank | 5.08 (4.62, 5.59) |  |
| **Setpoint** | Bimodal | 82.79 (79.17, 86.50) | 0.111 |
|  | Grating only | 82.18 (78.73, 85.89) |  |
|  | Odor only | 86.64 (82.53, 91.27) | 0.002 |
|  | Blank | 85.44 (81.35, 89.82) |  |
| **Curvature change** | Bimodal | 0.053 (0.039, 0.069) | 0.017 |
|  | Grating only | 0.047 (0.036, 0.061) |  |
|  | Odor only | 0.030 (0.022, 0.038) | 0.001 |
|  | Blank | 0.025 (0.019, 0.031) |  |

**Supplementary table 1.** Mean (95% CI) of average whisking amplitude, setpoint, absolute curvature change, both within and between sessions, presented in **Fig. 3**. Averages are computed over the 2 sec stimulus presentation epoch. P-values are from the statistical tests comparing bimodal versus grating only conditions and odor only versus blank (Mann-Whitney U test within sessions and Wilcoxon signed-rank test between sessions).

| **Within sessions** | | | |
| --- | --- | --- | --- |
|  | **Condition** | **Mean (95% CI)** | **p-value** |
| **Amplitude** | Bimodal | 0.33 (0.30, 0.37) | 1.99-8 |
|  | Grating only | 0.44 (0.40, 0.49) |  |
|  | Odor only | 0.33 (0.30, 0.26) | 1.45^-7^ |
|  | Blank | 0.30 (0.27, 0.34) |  |
| **Frequency** | Bimodal | 3.03 (2.94, 3.12) | 0.272 |
|  | Grating only | 3.07 (2.99, 3.15) |  |
|  | Odor only | 2.91 (2.84, 2.99) | 0.08 |
|  | Blank | 3.00 (2.91, 3.08) |  |
| **Between sessions** | | | |
| **Amplitude** | Bimodal | 0.33 (0.17, 0.58) | 0.002 |
|  | Grating only | 0.44 (0.21, 0.76) |  |
|  | Odor only | 0.33 (0.17, 0.57) | 0.002 |
|  | Blank | 0.44 (0.21, 0.74) |  |
| **Frequency** | Bimodal | 3.03 (2.84, 3.30) | 0.637 |
|  | Grating only | 3.07 (2.81, 3.34) |  |
|  | Odor only | 2.91 (2.69, 3.17) | 0.307 |
|  | Blank | 3.00 (2.75, 3.26) |  |

**Supplementary table 2.** Mean (95% CI) of average breathing amplitude and frequency, both within and between sessions, presented in **Fig. 3**. Averages are computed over the 2 sec stimulus presentation epoch. P-values are from the statistical tests comparing bimodal versus grating only conditions and odor only versus blank (Mann-Whitney U test within sessions and Wilcoxon signed-rank test between sessions).

| **Within sessions** | | | |
| --- | --- | --- | --- |
|  | **Condition** | **Mean (95% CI)** | **p-value** |
| **Amplitude** | Bimodal | 0.36 (0.32, 0.39) | 4.97^-6^ |
|  | Grating only | 0.44 (0.40, 0.49) |  |
|  | Odor only | 0.35 (0.32, 0.38) | 7.74^-5^ |
|  | Blank | 0.41 (0.37, 0.45) |  |
| **Frequency** | Bimodal | 3.37 (3.27, 3.48) | 0.5 |
|  | Grating only | 3.33 (3.23, 3.44) |  |
|  | Odor only | 2.97 (2.87, 3.08) | 0.52 |
|  | Blank | 2.94 (2.84, 3.04) |  |
| **Between sessions** | | | |
| **Amplitude** | Bimodal | 0.36 (0.19, 0.59) | 0.005 |
|  | Grating only | 0.44 (0.24, 0.74) |  |
|  | Odor only | 0.35 (0.18, 0.58) | 0.006 |
|  | Blank | 0.41 (0.22, 0.67) |  |
| **Frequency** | Bimodal | 3.37 (3.04, 3.77) | 0.507 |
|  | Grating only | 3.33 (3.02, 3.68) |  |
|  | Odor only | 2.97 (2.59, 3.42) | 0.721 |
|  | Blank | 2.94 (2.57, 3.36) |  |

**Supplementary table 3**. Same as Supplementary table 2 for the passive context.
