# Supplementary material for: Olfactory modulation of barrel cortex activity during active whisking and passive whisker stimulation
Source: Nat Commun. 2022 Jul 2;13:3830. doi: 10.1038/s41467-022-31565-0 (PMC9250522; doi:10.1038/s41467-022-31565-0)
Supplement: Supplementary file 3 — Reporting Summary [file 41467_2022_31565_MOESM3_ESM.pdf]

nature portfolio

Corresponding author(s): Brice Bathellier

Last updated by author(s): Jun 10, 2022

Reporting Summary

Nature Portfolio wishes to improve the reproducibility of the work that we publish. This form provides structure for consistency and transparency in reporting. For further information on Nature Portfolio policies, see our Editorial Policies and the Editorial Policy Checklist.

Statistics

For all statistical analyses, confirm that the following items are present in the figure legend, table legend, main text, or Methods section.

n/a	Confirmed	
☐	☒	The exact sample size (*n*) for each experimental group/condition, given as a discrete number and unit of measurement
☐	☒	A statement on whether measurements were taken from distinct samples or whether the same sample was measured repeatedly
☐	☒	The statistical test(s) used AND whether they are one- or two-sided<br>*Only common tests should be described solely by name; describe more complex techniques in the Methods section.*
☐	☒	A description of all covariates tested
☐	☒	A description of any assumptions or corrections, such as tests of normality and adjustment for multiple comparisons
☐	☒	A full description of the statistical parameters including central tendency (e.g. means) or other basic estimates (e.g. regression coefficient) AND variation (e.g. standard deviation) or associated estimates of uncertainty (e.g. confidence intervals)
☐	☒	For null hypothesis testing, the test statistic (e.g. *F*, *t*, *r*) with confidence intervals, effect sizes, degrees of freedom and *P* value noted<br>*Give P values as exact values whenever suitable.*
☒	☐	For Bayesian analysis, information on the choice of priors and Markov chain Monte Carlo settings
☒	☐	For hierarchical and complex designs, identification of the appropriate level for tests and full reporting of outcomes
☒	☐	Estimates of effect sizes (e.g. Cohen's *d*, Pearson's *r*), indicating how they were calculated

Our web collection on statistics for biologists contains articles on many of the points above.

Software and code

Policy information about availability of computer code

Data collection: Femtonics and Karthala system two-photon microscope data acquisition software, Matlab-based custom software for olfactory and tactile stimulation.

Data analysis: Suite 2P for 2-photon data analysis (https://www.suite2p.org/). Facemap for facial behavior. Both Suite 2P and Facemap do not provide version numbers. Custom Python 3 code freely available from the Zenodo database https://doi.org/10.5281/zenodo.6397722.

For manuscripts utilizing custom algorithms or software that are central to the research but not yet described in published literature, software must be made available to editors and reviewers. We strongly encourage code deposition in a community repository (e.g. GitHub). See the Nature Portfolio guidelines for submitting code & software for further information.

Data

Policy information about availability of data

All manuscripts must include a data availability statement. This statement should provide the following information, where applicable:

- Accession codes, unique identifiers, or web links for publicly available datasets
- A description of any restrictions on data availability
- For clinical datasets or third party data, please ensure that the statement adheres to our policy

Statement: "Data availability: The complete dataset supporting these findings is freely available from the Zenodo database https://doi.org/10.5281/zenodo.6397722."

Field-specific reporting

Please select the one below that is the best fit for your research. If you are not sure, read the appropriate sections before making your selection.

☒ Life sciences ☐ Behavioural & social sciences ☐ Ecological, evolutionary & environmental sciences

For a reference copy of the document with all sections, see nature.com/documents/nr-reporting-summary-flat.pdf

Life sciences study design

All studies must disclose on these points even when the disclosure is negative.

Sample size	Sample sizes (15>n>10) was determined to ensure validity and sensitivity of non-parametric tests for any consistent effect (i.e. effect observed in >80% of the samples).
Data exclusions	No data was excluded from the study
Replication	We performed the experiment 3 times with different groups of animal and once with another set of conditions (set of stimuli). All replications yielded similar conclusions.
Randomization	Stimulus presentations were randomized. We did not perform group comparisons so no randomizations of groups was necessary.
Blinding	No blinding was used. All analyses and experiments are done with automated softwares.

Reporting for specific materials, systems and methods

We require information from authors about some types of materials, experimental systems and methods used in many studies. Here, indicate whether each material, system or method listed is relevant to your study. If you are not sure if a list item applies to your research, read the appropriate section before selecting a response.

Materials & experimental systems

n/a	Involved in the study	
☒	☐	Antibodies
☒	☐	Eukaryotic cell lines
☒	☐	Palaeontology and archaeology
☐	☒	Animals and other organisms
☒	☐	Human research participants
☒	☐	Clinical data
☒	☐	Dual use research of concern

Methods

n/a	Involved in the study	
☒	☐	ChIP-seq
☒	☐	Flow cytometry
☒	☐	MRI-based neuroimaging

Animals and other organisms

Policy information about studies involving animals; ARRIVE guidelines recommended for reporting animal research

Laboratory animals	We used 8 to 12-weeks-old C57BL/6J male and female mice housed 1-4 per cage, in normal light/dark cycle (12h/12h).
Wild animals	We did not use wild animals in this study
Field-collected samples	We did not use field-collected samples
Ethics oversight	French Ethical Committees #59 and #89 (authorizations APAFIS#9714-2018011108392486 v2 and APAFIS#27040-2020090316536717 v1)

Note that full information on the approval of the study protocol must also be provided in the manuscript.
